# What Long COVID Prevention Strategies Suggest About Its Pathophysiology

**DOI:** 10.1093/ofid/ofad466

**Published:** 2023-09-08

**Authors:** Lao-Tzu Allan-Blitz, Howard Hu, Jeffrey D Klausner

**Affiliations:** Division of Global Health Equity, Department of Medicine, Brigham and Women's Hospital, Boston, Massachusetts, USA; Department of Population and Public Health Sciences, Keck School of Medicine, University of Southern California, Los Angeles, California, USA; Department of Population and Public Health Sciences, Keck School of Medicine, University of Southern California, Los Angeles, California, USA

To the  Editor—Despite a high burden of disease, what drives long COVID remains unknown; however, recent epidemiologic work describing a reduction in risk of long COVID attributable to several interventions (Table 1) [1–11] may shed light on the pathophysiology and potential preventive strategies. Metformin was shown in a large randomized trial to reduce the odds of long COVID by >40% when given within 7 days of symptom onset [1], while nirmatrelvir/ritonavir, when given within 5 days of a positive SARS-CoV-2 test result, was shown in a cohort study to reduce the risk of long COVID by 26% [2]. Finally, a meta-analysis of 12 observational studies demonstrated that even a single immunization against SARS-CoV-2 prior to infection reduced the odds of long COVID, with increasing protection conferred by additional doses [12].

**Table 1.** Change in Risk of Long COVID due to Various Outpatient Treatments and Immunizations

First Author	Year	Country	No. of Participants	Intervention		Risk of Long COVID (95% CI)
Bramante	2023	USA	1126	Metformin		HR, 0.59 (.39–.89)
Xie	2023	USA	281 793	Nirmatrelvir/ritonavir		RR, 0.74 (.72–.77)
Uehara	2023	Japan	666	Ensitrelvir^a^	125 mg (n = 232)	25% reduction
					216 mg (n = 216)	10% reduction
Al-Aly	2022	USA	5 017 431	Vaccination^b^	2 doses	HR, 0.85 (.82–.89)
Emecen	2023	Turkey	5610	Vaccination^c^	2 doses	OR, 0.53 (.40–.72)
Azzolini	2022	Italy	2560	Vaccination	3 doses (BNT162b2)	OR, 0.16 (.03–.84)
					2 doses (BNT162b2)	OR, 0.25 (.07–.87)
					1 dose (BNT162b2)	OR, 0.65 (.44–.98)
Ayoubkhani	2022	UK	28 356	Vaccination^d^	2 doses	OR, 0.91 (.86–.97)
					1 dose	OR, 0.87 (.81–.93)
Ayoubkhani	2022	UK	3090	Vaccination^d^	2 doses	OR, 0.59 (.50–.69)
Antonelli	2022	UK	1 240 009	Vaccination^d^	2 doses	OR, 0.51 (.32–.82)
Simon	Preprint	USA	240 648	Vaccination^e^	1 dose	OR, 0.22 (.20–.25)
Senjam	Preprint	India	773	Vaccination^f^	2 doses	OR, 0.65 (.45–.96)

Abbreviations: HR, hazard ratio; OR, odds ratio; RR, risk ratio.

a Unpublished data from phase 3 of a phase 2/3 multicenter trial.

b Vaccination defined as 14 days after (1) second dose of mRNA-1273 or BNT162b2 or (2) first shot of Ad26.

c Vaccination was defined among those 14 days after the second dose of CoronaVac or BNT162b2.

d Vaccination was with mRNA vaccine (BNT162b2 or mRNA-1273) or AZD1222.

e Vaccination was defined as any of the following: mRNA-1273, BNT162b2, or Ad26.

f Type of vaccine not specified.

Some hypothesize that long COVID is the result of persistent viral antigens [13], resulting in ongoing immune dysregulation or immune-mediated organ damage. Were that to be the case, however, we would not expect to see the protective effects of therapies provided *early* in the disease course. Metformin and nirmatrelvir/ritonavir reduce the risk of long COVID when administered within the first week of a positive test result, and the protective effect of immunization is mostly clearly demonstrated when given prior to infection. We suspect that long COVID is driven by conditions that arise early on.

While nirmatrelvir/ritonavir and prior immunization have established direct anti–SARS-CoV-2 properties, metformin may have direct and indirect anti–SARS-CoV-2 effects [14]. Prior work has demonstrated that metformin suppresses SARS-CoV-2 growth *in vitro* and in *ex vivo* lung tissue [15, 16]. Metformin may also abrogate SARS-CoV-2 nonstructural protein 6–mediated lysosomal deacidification, thereby restoring autophage flux [17]. Restoration of autophage flux, in turn, may reduce consequent inflammation [17], facilitate viral clearance, and reduce the effectiveness of viral replication [18].

It is notable, then, that all of the strategies effective in reducing the risk of long COVID might affect SARS-CoV-2 viral load during early infection, directly or indirectly. We therefore hypothesize that long COVID can be predicted by the peak and duration of the SARS-CoV-2 viral load—the area under the viral load curve during initial infection. The consequence of that hypothesis is that long COVID might be prevented by aggressively reducing the initial SARS-CoV-2 viral load.

Our hypothesis is analogous to the set point hypothesis in early HIV infection. The set point hypothesis argues that high early viral loads are associated with a higher subsequent viral nadir, or set point, which has been associated with more rapid clinical deterioration and progression to AIDS [19]. Importantly, the HIV viral set point is also dramatically reduced by early antiretroviral therapy initiation [20].

Our hypothesis is supported by the results of a phase 3 clinical trial evaluating the impacts of ensitrelvir (a protease inhibitor) on acute SARS-CoV-2 infection and long COVID. Ensitrelvir demonstrated marked reductions in viral loads early in the course of infection [21] and was associated with a reduction in the risk of long COVID [3]. Much more work is needed, however, to investigate that hypothesis. Subsequent evaluation of pharmacotherapeutic prevention of long COVID should assess viral loads during early infection. Additionally, follow-up of patients from earlier clinical trials may provide prospective data comparing the incidence of long COVID by initial viral loads. Trials such as the Evaluation of Protease Inhibition for COVID-19 in Standard-Risk Patients collected baseline viral load data among patients treated with nirmatrelvir/ritonavir [22]. Importantly, given the heterogeneity of long COVID presentations, it will be important for public health officials to develop a standardized case definition that can be used across studies.

Should long COVID be dependent on the initial peak and duration of SARS-CoV-2 viral load, one strategy for combating long COVID would then entail aggressively reducing viral loads during initial infection. If our hypothesis is supported by more definitive studies, indications for SARS-CoV-2 therapeutics might be liberalized. Our messaging to the public would encourage individuals to continue testing and seeking early antiviral treatment with the aim of preventing long COVID. While much of our focus until now has rightly been on addressing acute SARS-CoV-2 infection, with the waning severity of disease comes an opportunity to address and potentially prevent the long-term consequences of long COVID.

## Notes

***Patient consent statement.*** No human subjects research was conducted as the letter was based on previously published reports.
